# Microbial Community Analysis and Food Safety Practice Survey-Based Hazard Identification and Risk Assessment for Controlled Environment Hydroponic/Aquaponic Farming Systems

**DOI:** 10.3389/fmicb.2022.879260

**Published:** 2022-05-19

**Authors:** Mengyi Dong, Hao Feng

**Affiliations:** Department of Food Science and Human Nutrition, University of Illinois at Urbana-Champaign, Urbana, IL, United States

**Keywords:** hydroponic, microgreen, microbiome, lettuce, pathogen identification, system health

## Abstract

Hydroponic and aquaponic farming is becoming increasingly popular as a solution to address global food security. Plants in hydroponic systems are grown hydroponically under controlled environments and are considered to have fewer food safety concerns than traditional field farming. However, hydroponics and aquaponics might have very different sources of microbial food safety risks that remain under-examined. In this study, we investigated the microbiomes, microbial hazards, and potential bacterial transmission routes inside two commercial hydroponic and aquaponic farming systems using 16S-ITS-23S rRNA sequencing and a hydroponic food safety practice survey. The hydroponic farming system microbiome was analyzed from the fresh produce, nutrient solution, tools, and farmworkers. Proteobacteria, Actinobacteria, Cyanobacteria, Bacteroidetes, and Firmicutes were the main components of hydroponic/aquaponic farming systems, with *Pseudomonas* being the most abundant genus in fresh produce samples. We further identified the presence of multiple spoilage bacteria and potential human, plant, and fish pathogens at the subspecies level. Spoilage *Pseudomonas* spp. and spoilage *Clostridium* spp. were abundant in the hydroponic microgreen farm and aquaponic lettuce farm, respectively. Moreover, we demonstrated the mapping of *Escherichia coli* 16s-ITS-23s rRNA sequence reads (∼2,500 bp) to small or large subunit rRNA databases and whole-genome databases to confirm pathogenicity and showed the potential of using 16s-ITS-23s rRNA sequencing for pathogen identification. With the SourceTracker and overlapping amplicon sequence variants, we predicted the bidirectional transmission route between plants and the surrounding environment and constructed the bacteria transmission map, which can be implemented in future food safety risk control plans.

## Introduction

Hydroponic cropping systems (HCSs) grow plants in a mixture of water and nutrient solutions in an enclosed and controlled environment (Riggio et al., 2019). Aquaponics is a form of HCS that utilizes water from a fish tank as the nutrition source for the plants. Compared with soil-based farms, the HCS is not restricted by the climate or location, better utilizes vertical spaces, saves approximately 90% irrigation water, and can supply fruits and vegetables to the surrounding communities year-round (Pegasus Agriculture Group, 2017). HCS is viewed as a promising solution for feeding the global growing population. The value of the global hydroponics market was estimated at $6.63 billion in 2016 and is projected to reach $12.1 billion in 2025 (FMR, 2019).

Hydroponic cropping system is considered to have fewer food safety issues because it eliminates the common field microbial contaminants from soil, surface water, wild and farm animals, and pests. It is also free from chemical contaminants such as pesticides and soil fertilizers (Center for Disease Control and Prevention [CDC], 2019; Morgan, 2019). However, there are several reported foodborne outbreaks and recalls related to the fresh produce grown in HCSs (Center for Disease Control and Prevention [CDC], 2014, 2016, 2020). The implementation rules from the United States Food and Drug Administration in response to the Food Safety Modernization Act require farmers to follow farm food safety plans and hazard analysis, and periodically engage in food safety audits (U.S. Food and Drug Administration, 2011). However, small, local, and direct-market farms such as most hydroponic/aquaponic farms were excluded from these regulations (Deering, 2018). Thus, there is an urgent need to analyze the food safety hazards and risks for these farms (Colorado Department of Public Health and Environment, 2012; Deering, 2018).

Orozco and Iturriaga (2008) detected *Salmonella* and *Escherichia coli* in a hydroponic tomato farm and attributed it to flood and wild birds entering the facility. Lopez-Galvez et al. (2014) reported the detection of *Salmonella* spp., *Listeria* spp., and *E. coli* in a hydroponic farm irrigated with reclaimed and surface water. There were also debates that pathogens that may present in fish feces would contaminate the fresh produce growing in aquaponic systems (Bergšpica et al., 2020; Sawyer et al., 2020). However, previous sporadic studies only reported incidents where contamination by pathogens was identified in HCSs, and no effort was made to systematically analyze the source and transmission of hazards.

Microbiomes in an ecosystem would provide insight into potential contamination sources and transmission (De Filippis et al., 2021). 16S rRNA sequencing with the operating taxonomic units classification method, as the major approach (Westcott and Schloss, 2015; Kopylova et al., 2016; Ranjan et al., 2016), has been used to characterize microbiomes related to agricultural and food systems, including soil, fresh produce surfaces, kitchen environment, and human hand samples (Fierer et al., 2008; Bibby et al., 2010; Flores et al., 2013; Leff and Fierer, 2013). Full-length 16S rRNA sequencing could distinguish bacteria at the species level; however, it is insufficient for subspecies-level hazard identification, for example, distinguishing closely related pathogenic and non-pathogenic *E. coli* (Srinivasan et al., 2015; Miao et al., 2017; Callahan et al., 2019; Deguenon et al., 2019; Numberger et al., 2019). To identify pathogen hazards, researchers usually target common pathogenic bacteria or specific virulence genes using selective culture or multiplex real-time PCR; however, adding multiple target gene primers is costly, and the targeted pathogens may not present in the tested samples (Lopez-Galvez et al., 2014; Liu et al., 2019; Dankwa et al., 2020; Zhong et al., 2020).

To improve the resolution and confidence of taxonomic classification, a novel amplicon sequencing approach was proposed, in which the full-length 16S rRNA, internal transcribed spacer (ITS), and partial 23S rRNA (16S-ITS-23S rRNA) was sequenced, resulting in a long read of ∼2,500 bp (Martijn et al., 2019; Graf et al., 2021; Kinoshita et al., 2021). This sequencing method can be performed on PacBio systems at a cost similar to the full-length 16S rRNA sequencing. Also, a new concept of amplicon sequence variants (ASVs) was developed to replace the OTU clustering and increase the classification resolution (Callahan et al., 2016, 2017). This approach has been used to distinguish closely related *Klebsiella*, *E. coli*, and *Enterobacter* strains in infant feces (Graf et al., 2021).

On the other hand, food safety practice survey has been used as a risk assessment tool for identifying potential farm food safety hazards (Soon et al., 2013; Ilic et al., 2017). Researchers have tried combining survey and sampling data from different studies to assess microbial food safety and public health risks (Allende et al., 2017; Barragán et al., 2021). Thus, the combination of a survey with microbiome sampling may provide a better understanding of the quantitative microbial measurements for the design of food safety risk reduction strategies.

This study was performed to explore the plant and environmental microbiomes in hydroponic cropping ecosystems and the influence of farming practice on the microbiomes with a combined usage of farming food safety practice survey and high throughput 16S-ITS-23S sequencing microbiome analysis. We compared the aerobic bacteria amount and microbiome composition and diversities of commercial hydroponic/aquaponic farms to laboratory control systems. Furthermore, we screened the microbiomes for microbial hazards, including plant, human, and fish pathogens and spoilage microorganisms, and proposed potential bacteria transmission routes inside the HCSs. Together with the survey response, we showcased the influences of farming food safety practices on environmental and plant microbiomes and provided customized or targeted improvement strategies for the farms.

## Materials and Methods

### Study Design and Recruitment

The recruitment and sampling were conducted from March to August 2020. Because the survey and sampling plan involved human subjects, an Institutional Review Board (IRB) approval for human studies was obtained from the University of Illinois Office for the Protection of Research Subjects (IRB protocol: 20653).

A total of 12 hydroponic farms in Illinois were identified by searching on Google Map and agriculture newsletters and the farms were contacted by phone calls or emails. Two farms participated in this study, including a vertical hydroponic microgreen farm (farm H) and an aquaponic farm that grows lettuce and tilapia together (farm A). The hydroponic system setup and operation procedures were present in Supplementary Figure 1. In farm A, the seeds were started in seed starting trays until seedlings were developed, then transferred to a flood-and-drain system until the baby lettuce rosette formed, then rosettes were transferred to a deep-water-culture (DWC) system for maturation. The water came from tilapia fish tanks, first supplied into the DWC system, then the flood-and-drain system and seed starting trays. Three lettuce cultivars were grown in the DWC system in the order of HoneyCrisp, Green Oak Leaf, and GreenCrisp from the inlet to the outlet. Farm H used two separated flood-and-drain systems and five-layer vertical structures. The first system grew five microgreen species, including garnet, radish, broccoli, cilantro, and brussels sprouts. The second system only had kale microgreens.

### Sampling Plan

The two farms were asked for consent on the sampling plan, farm visit, and survey. Fresh produce samples were collected from both farms. The samples were harvested directly from the growth trays with sterile gloves and scissors and weighed into sterile sample bags (Whirl-Pak, Madison, WI, United States). From farm H, radish, garnet, broccoli, brussels sprouts, cilantro, and kale microgreens were sampled by randomly harvesting from 10 different growth trays and added up to 25 *g* total weight each. We also grew broccoli, radish, and kale microgreens hydroponically in the laboratory system (L1) as a control. From farm A, three lettuce cultivars at their fully grown stage were randomly sampled (three heads each cultivar) from the DW system. Four similar hydroponically grown lettuce cultivars were purchased from a grocery store in Champaign, IL, for comparison. We grew romaine and oak leaf lettuce in a flood-and-drain system and a smaller DWC hydroponic system in the laboratory (L2) as a control.

Nutrient solution samples (250 ml each) were collected from both farms directly into sterilized sample bags. From farm H, we collected two nutrient solution samples from each flood-and-drain system at the reservoir tank and the tubing system underneath the sampled microgreen trays, respectively. Three nutrient solution samples were taken from the DWC of farm A at the water inlet from the fish tank, the midpoint of the system near lettuce roots, and the end of the system near the lettuce roots, respectively.

Environmental swabs were obtained from agreed sampling items using sterile cotton swabs. The swabs were presoaked in peptone–saline water for 1 min, and 3 × 3-cm areas were carefully swabbed with the entire area of the cotton swab surface. Three swab samples were obtained from farm H, including worker’s hands, shoe soles, and sanitized growth trays. Three swab samples were obtained from farm A, including growth tray swabs from the DWC system (trayM), the flood-and-drain system (trayB), and shoe sole swab from the farmworker.

All collected samples were transferred to a microbiology laboratory at the University of Illinois in a portable cooler on the same day. The samples collected for microbiome analysis are listed in Supplementary Table 1.

### Survey Design

A five-section survey was developed focusing on current farm food safety practices, including worker health and hygiene, food safety awareness, irrigation water treatment, equipment sanitation, and produce handling procedures (Supplementary Table 2). The questions were gathered from available farm food safety surveys with modifications to reflect the production practices of the hydroponic systems (Harrison et al., 2013; Ilic et al., 2017; FamilyFarmed, 2019). To ensure privacy, each farm was assigned a code to link back to the sampling data. The survey result was coded into numeric scores for calculating the total scores for each section.

### Microbial Load Enumeration

The aerobic plate counts were performed for fresh produce and nutrient solution samples. A 25 *g* of the fresh produce samples were mixed with 225 mL of phosphate–buffered saline in sterilized, filtered sample bags. After homogenizing for 2 min in a stomacher and series dilution, 100 μL filtrates of the fresh produce samples or nutrient solution samples were spread onto plate count agar (BD Difco, Franklin Lakes, NJ, United States). The plates were incubated at 37 ± 2°C for 24 h and the colony-forming units (CFU) were counted. The remaining filtrates and nutrient solution samples were filtered using 0.2 μm-pored 250 mL vacuum filters (Foxx Life Sciences, Pittsburgh, PA, United States) to collect the microorganisms. The swab samples and filters were stored at –20°C for less than 2 weeks before molecular analysis.

### DNA Isolation and 16S-ITS-23S rRNA Gene Sequencing

To profile the microbiome, bacterial DNA was extracted from the swabs and filters using the DNeasy PowerSoil Pro kit (QIAGEN, Hilden, Germany), following the manufacturer’s instructions. Positive control with 12 known bacterial strains was extracted together with the regular samples. The DNA quality was evaluated by gel imaging, and concentration was determined using the Qubit dsDNA HS Assay Kit and Qubit 2.0 Fluorometer (Invitrogen, Thermo Fisher Scientific, Oregon, United States). The extracted DNA samples were stored at –20°C before further analysis.

The Wave StrainID kit (Shoreline Biome, Farmington, CT, United States) was used to sequence the amplicon that spans the full-length 16S, ITS, and partial 23S rRNA genes. The procedures were previously described by Graf et al. (2021). Amplicon libraries were created using the SMRTbell express template prep kit 2.0 (catalog number 100-938-900; Pacific Biosciences, Menlo Park, CA, United States) according to the manufacturer’s instructions. The library was sequenced on 1 SMRTBell 8M on a PacBio Sequel II system (Pacific Biosciences) using the circular consensus (CCS) sequencing mode at the University of Illinois Roy J. Carver Biotechnology Center, Urbana, IL, United States. The circular consensus reads (ccs) were determined with a minimum predicted accuracy of 0.999 and the minimum number of passes set to three in the SMRT Link software package 5.1 (Pacific Biosciences). A total of 826,974 ccs reads with a mean read length of 2,421 bp were produced using default settings.

### Sequence Processing and Taxonomic Assignment

The ccs were further processed using SBanalyzer 2.4 (Shoreline Biome) following the workflow described by Graf et al. (2021). Briefly, all reads were sorted into FASTQ files by sample with no trimming and classified by mapping to the Athena database (Shoreline Biome). The taxonomic assignments were made at >97% identity for strain level and >95% for species-level matched with reference 16S-ITS-23S rRNA sequence in the Athena database. After demultiplexing, the ccs were further processed with DADA2 (version 1.9.1) to obtain amplicons with single-nucleotide resolution (Callahan et al., 2019). ASVs were assigned eight taxonomy levels: kingdom, phylum, class, order, family, genus, species, and subspecies. The taxonomic ID and the corresponding read count for all samples were created at the end resulting in a total of 1,939 ASVs. Additionally, contaminant ASVs were detected and removed with the R package “decontam” using a prevalence-based contaminant identification with a threshold value cutoff of 0.5 (Davis et al., 2018). After decontamination, 1,096 ASVs remained.

### Microbial Community Analysis

Microbial community analysis was performed using the “Bioconductor,” “microbiome,” “phyloseq,” and “vegan” packages and visualized with ggplot2 in R (Gentleman et al., 2004; McMurdie and Holmes, 2013; Oksanen et al., 2020; Wickham; Lahti and Shetty). The dataset was transformed compositionally to visualize the phylum-level compositions and the 50 most abundant ASV genera. An untransformed dataset was used for community diversity analysis. Alpha-diversity indexes “Chao1,” “Shannon,” and “Simpson” were calculated. The normal distribution of individual alpha-diversity indexes was tested with the Shapiro–Wilk normality test. The data were evaluated for homogeneity of variance using Levene’s test. The statistical significance of alpha-diversity was evaluated using a repeated-measures ANOVA followed by Tukey’s test. The alpha-diversities were also compared by sample types or sampling location using a pairwise *t*-test with Benjamini–Hochberg procedure to reduce the false discovery rate. Beta diversity was visualized using principal coordinate analysis (PCoA) plots with phylogenetic-based weighted and unweighted UniFrac distance, as well as count-based Bray–Curtis distance. Permutational Multivariate Analysis of Variance (PERMANOVA) analysis was performed using the “adonis” function to measure the differences in beta diversity indexes by sampling location or type (Anderson, 2017).

### Identification of Pathogen and Spoilage Species

The presence of pathogens in all samples was screened against the NCBI pathogen database (NCBI, 2021). The spoilage bacterial species associated with fresh produce were screened (Kaczmarek et al., 2019). The spoilage ASVs were grouped by species or genus. The reads were transformed compositionally to percentage values and visualized as heatmaps using the ‘‘pheatmap’’ function^1^.

ASV1628 was marked as “*E. coli* unclassified” and it contains 38 sequence reads. We used it as an example to further examine potential pathogenicity. The 38 seed sequences were mapped against the SILVA databases with EMBL-EBI/ENA, GTDB, RDP, LTP taxonomy, using the SILVA Alignment, Classification, and Tree Services (ACT) to small subunit (16S) and large subunit (23S), respectively (Leinonen et al., 2011; Pruesse et al., 2012; Quast et al., 2013; Cole et al., 2014; Yilmaz et al., 2014; Chaumeil et al., 2020). The 38 seed sequences were also mapped against the bacterial whole-genome sequences in NCBI nucleotide collection (nt) using the Basic Local Alignment Search Tool (BLASTn; Zhang et al., 2000; Morgulis et al., 2008). For each seed sequence, the aligned sequences of the 10 most closely related strains with the highest identities were fetched and fast minimum evolution trees were constructed (Rzhetsky and Nei, 1993). The closely related pathogenic strains were screened and aligned with seed sequences using ClustalW in Jalview 2 (Version 2.11.1.4; Larkin et al., 2007; Waterhouse et al., 2009). After alignment, the Jukes–Cantor distances between seed sequence and fetched sequences were calculated using MegaX and with gamma parameter = 1 (Jukes and Cantor, 1969; Stecher et al., 2020). Potential pathogens are those within a 0.03 Jukes–Cantor distance of a known pathogen sequence (Ibekwe et al., 2013).

### SourceTracker and Shared Amplicon Sequence Variants

Microbial source tracking was achieved using the SourceTracker (version 1.0.1) R package with default parameters (Knights et al., 2011). The sequencing depth at 1,000 sequences/sample was chosen according to Zwirzitz et al. (2020) that 1,000 is adequate to provide a comparable result to deeper sequenced datasets. The nutrient solution and environmental samples were assigned as sources, and the fresh produce samples were assigned as sinks. The outputs were visualized using the Sankey flow diagram^2^. The shared ASVs between different system samples were visualized using the Venn diagram^3^.

## Results

### Bacterial Cell Counts From Different Sampling Locations and Sample Types

**FIGURE 1:**
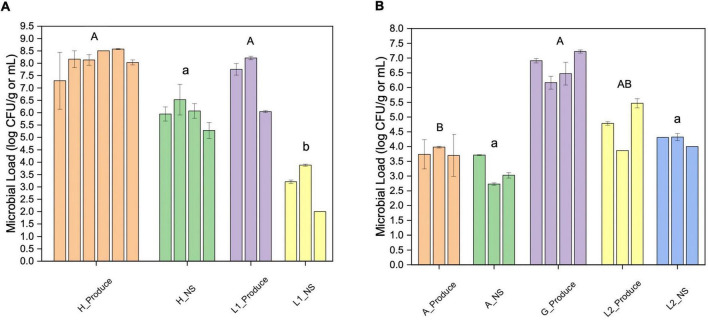
Aerobic plate counts of fresh produce and their nutrient solutions from different sampling locations. **(A)** Microgreen (Produce) and the nutrient solution (NS) from farm H and laboratory system 1 (L1); **(B)** lettuce (Produce) and the nutrient solution (NS) from farm A, grocery store (G), and lab system 2 (L2). The error bar indicates the standard deviation (SD). Different upper or lower-case letters indicate significant differences between sample groups (*p* < 0.05).

The overall microbial loads were quantified using aerobic plate counts. The microgreens from the hydroponic farm (farm H) and the laboratory system (L1) showed similar bacterial loads, ranging from 7.3 to 8.6 log CFU/g (Figure 1A). The lettuce samples from the aquaponic farm (farm A) had significantly lower bacterial counts (3.7–4.0 log CFU/g) than the lettuce samples from the grocery store (G, Figure 1B).

The farm H nutrient solution showed significantly higher bacterial loads than that of the L1. The bacterial loads of farm A nutrient solution were significantly lower than that of the laboratory hydroponic systems (L2). In farm A water system, the highest bacteria count was detected at the inlet from the fish tank (3.7 log CFU/mL).

### Food Safety Practice Survey

The food safety awareness and practice of two farms were surveyed, and the scores are shown in Supplementary Table 3. Farm H implemented overall better food safety practices than farm A and received higher scores in equipment and environment sanitation and food safety awareness, such as sanitizer application and usage of personal protection equipment. Both farms paid minimum attention to the treatment of circulating water, which may result in the accumulation of waste and microbial hazards in the system. In fresh produce handling, farm A used tap water to rinse the seeds before starting germination, while farm H did not implement any seed treatment.

### Microbial Community Structure and Relationship

**FIGURE 2:**
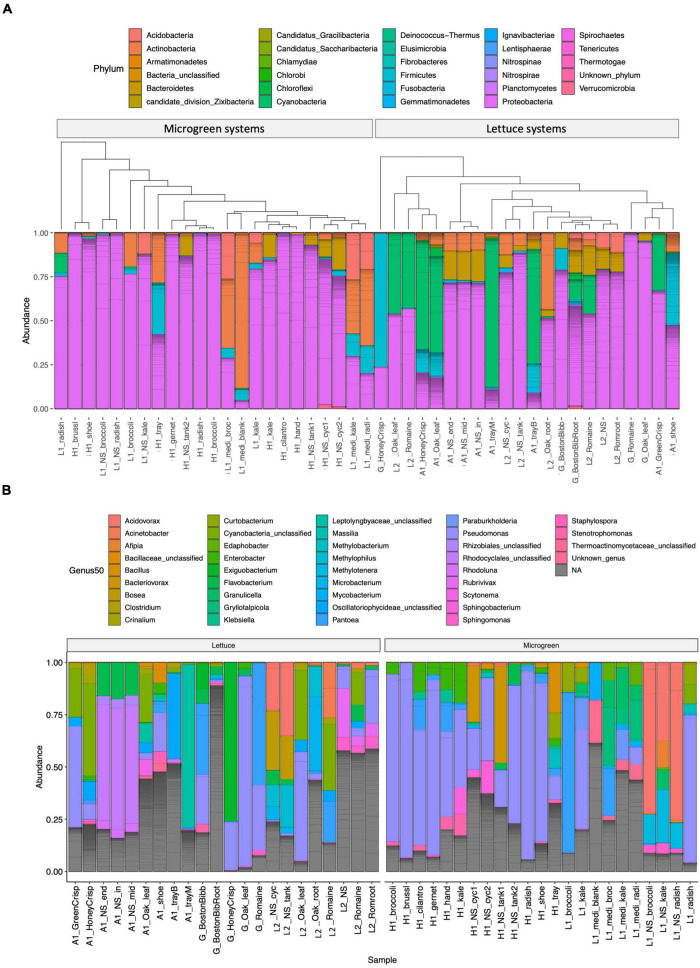
Microbiome compositions. **(A)** Phylum-level composition: samples are grouped by microgreen (left) or lettuce (right) systems. The samples are clustered by microbiome similarity using the phylogenetic tree structures. **(B)** Genus-level classification of the 50 most abundant ASVs parted by system type of lettuce (left) or microgreen (right). Individual ASVs are separated by a gray line within the bar graphs.

The microbiome compositions and the genera of the 50 most abundant ASVs are displayed in Figure 2 and Supplementary Table 4. Proteobacteria was the most abundant group in microgreens systems (Figure 2A). *Pseudomonas* was the dominant genus in the farm H, and *P. alcaligenes* (ASV1746), *P. fluorescens* (ASV1756 and ASV1758), and *P. lutea* (ASV1767) were among the 50 most abundant ASVs. Actinobacteria and firmicutes are resistant to sanitation treatments (Ramlal et al., 2021). They were both presented in the blank microgreen growth medium, sanitized trays, and on workers’ shoes. Cyanobacteria are the common photosynthetic bacteria in wastewater and are known as “blue-green algae.” They were commonly observed in lettuce systems (farm A and L2) as green substances on the growth trays and near plant roots. Five cyanobacteria ASVs were identified within the 50 most abundant ASVs. Cyanobacteria over-grow would produce hazardous toxins and deplete oxygen, and cause aquatic animal death (Ezenarro et al., 2021).

The microbiomes from the same sample type and sampling location were arranged closely on the tree (Figure 2A). Samples of similar types from different locations were also closely clustered with each other, such as the lettuce sample from the grocery store and the L2, and the kale microgreens from the farm H and L1.

### Microbiome Diversities as Affected by Location and Sample Type

**FIGURE 3:**
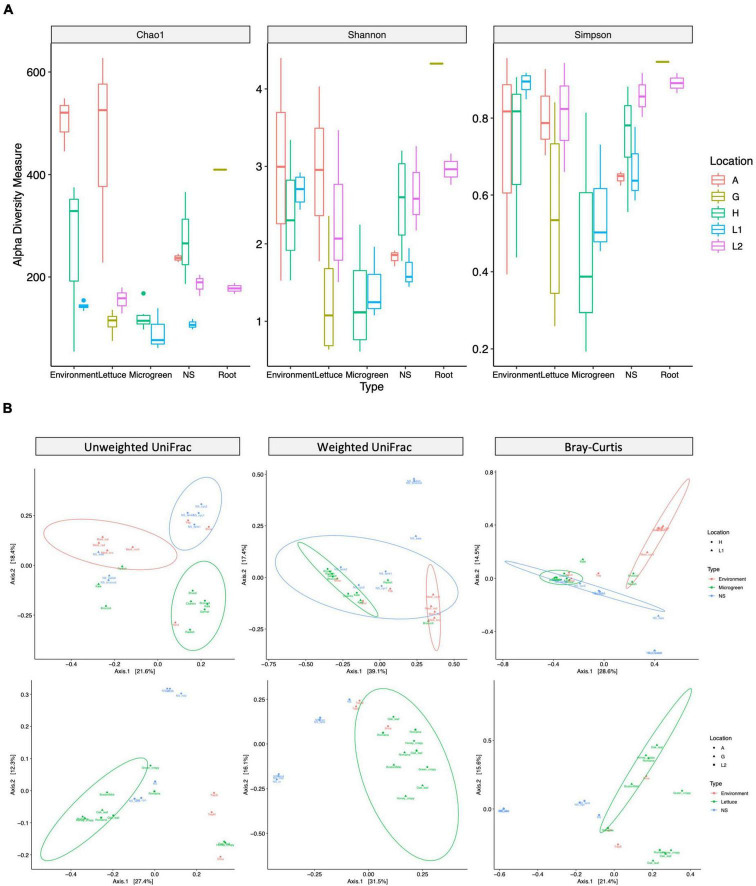
Alpha- and beta-diversity measurements of bacteria communities. **(A)** Alpha-diversity: the community richness and evenness of different sample types. Alpha-diversity index: chao1 measures community richness; Shannon and Simpson measure community richness and evenness. The box represents the first and third quartiles, the center line represents the median, and whiskers as min-to-max values. **(B)** Beta-diversity: microbiome compositional difference between sample types. Samples are grouped by microgreen (top row, H and L1) or lettuce (bottom, A, G, and L2) systems. Unweighted UniFrac (left, presence of taxa), weighted UniFrac (middle, presence and relative abundance of taxa), and Bray–Curtis (right, presence and relative abundance of taxa) distance are visualized by PCoA plots. The ellipses indicate 95% confidence regions for clusters by the normal distribution.

The microbiome diversities were examined using multiple alpha-diversity (Figure 3A) and beta-diversity indexes (Figure 3B). The microgreens from farms H and L1 had similar community alpha-diversities. However, the nutrient solutions from farm H had higher community richness than that of L1. Farm H nutrient solution was shared by multiple microgreens, potentially adding to the community richness. The lettuce samples and environmental samples (shoe and tray swabs) from farm A showed the highest community richness among all samples (Figure 3A).

Permutational Multivariate Analysis of Variance test revealed significant compositional differences between different sample types and different sampling locations (Supplementary Table 5). According to the unweighted UniFrac distance, farm H microgreens had similar ASVs with worker’s hand, while its nutrient solutions had similar ASVs with shoes and tray; two lettuce samples from farm A had similar ASVs with worker’s shoes instead of other lettuce (Figure 3B left). Microgreen and lettuce samples from different locations clustered together on the weighted UniFrac plot, indicating plant microbiomes from different sampling sites had shared phylogenetic patterns (Figure 3B middle).

### Pathogen and Spoilage Organism Identification

**FIGURE 4:**
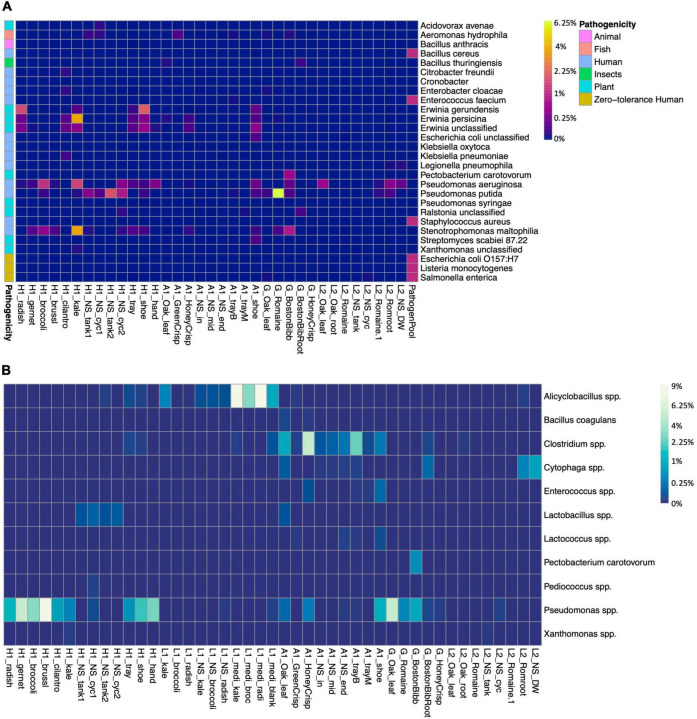
Presence of pathogens and bacteria of interest, and spoilage species in samples. **(A)** Presence of pathogens and bacteria of interest. Colored blocks on the left side indicate the potential pathogenicity toward animal, fish, human, insects, or plants. L1 samples are free of any listed strains, thus not shown in the graph. **(B)** Presence of spoilage bacteria species in samples, the spoilage-related ASVs were grouped at the species level. The heatmap color scale is in square root scale.

We screened the microbiomes for the presence of plant, human, and fish pathogens as well as spoilage organisms (Figure 4). The zero-tolerance human pathogen *E. coli* O157:H7, *Salmonella* spp., and *Listeria monocytogenes* were not identified in either farm. An unclassified *E. coli* (ASV1628) was presented on farm A worker’s shoes (Figure 4A). *E. coli* is used as an indicator for fecal contamination in field farming (Kaczmarek et al., 2019). According to the survey, farm A had an outdoor farm adjunct to the greenhouse, and the workers did not switch shoes between farms. Even though a sanitizer sink was presented at the entrance, it failed to eliminate the risk of bringing in contaminants from the open environment. *Pseudomonas aeruginosa* is a human pathogen and was abundant in farm H kale and broccoli microgreens, L2 lettuce, and on farm A worker’s shoes. Plant pathogen *P. syringae* was identified in low abundance on the farm H worker’s shoes. *P. syringae* is known to cause bacterial leaf spots on multiple cruciferous microgreen species (UMass Extension, 2018). *P. putida* is a beneficial organism for promoting plant growth but also an opportunistic human pathogen, and it was abundant in farm H nutrient solution. The fish pathogen *Aeromonas hydrophila* (ASV1571 and ASV1572) was identified in low abundance in farm A GreenCrisp lettuce but not in other samples (Figure 4). If water was circulated between the plant system and the fish tank without pretreatments, there would be a potential risk of pathogen transmission *via* water to fish.

Spoilage bacteria would deteriorate foods and generate unpleasant odor, taste, and texture. The major fresh produce spoilage is by lactic acid bacteria, and this group includes *Lactobacillus*, *Leuconostoc*, *Pedicoccus, Lactococcus*, and *Enterococcus* (Kaczmarek et al., 2019). *Lactobacillus*, *Lactococcus*, and *Enterococcus* were identified in multiple samples of farm A and nutrient solution of farm H (Figure 4B). The *Pseudomonas* spp. were abundant in farm H microgreens and on the sanitized trays, farm A shoes and lettuce, and G lettuce, including spoilage species of *P. fluorescens*, *P. viridiflava*, and *P. tolaasii*. Farm H had two systems and according to the survey, system 1 nutrient solution (H1_NS_tank1 and H1_NS_cyc1) had been circulated for almost 1 month, while system 2 nutrient solution (H1_NS_tank2 and H1_NS_cyc2) was recently changed. The spoilage *Pseudomonas* spp. was relatively lower in system 2 nutrient solution and kale microgreens compared to system 1 and the other five microgreens. The genus *Clostridium* was identified in farm A samples, and it included multiple spoilage species. However, the foodborne outbreak causing species *C. botulinum* and *C. perfringens* were not detected. The farm A worker’s shoes reserved multiple spoilage bacteria, again, indicating inadequate sanitation.

### *Escherichia coli* Amplicon Sequence Variants Sequences Mapped to Multiple Databases

The pathogenicity of ASV1628 (“*E. coli*_unclassified”) was uncertain. Thus, we further mapped the 38 sequences in ASV1628 against multiple rRNA sequence databases (Supplementary Table 6). The large subunit (23S) databases (mean identity 93.2%) classified all 38 sequences as *Escherichia–Shigella* with 84 neighbor strains having >97% identity and 9 neighbor *E. coli* strains aligned 100% with the seed 23S partial sequence. Better identities were achieved using small subunits (16S) databases (mean identity 99.1%) with 130 neighbor strains having >97% identity, and some seed sequences were classified as *Enterobactor*, *Pantoea*, or *Salmonella*. We were not able to increase taxonomy resolution with small subunit or large subunit rRNA databases.

To further confirm the pathogenicity, we mapped 38 seed sequences against the whole genome data in NCBI and calculated the Jukes–Cantor distance between seed sequences and selected pathogenic strains (Supplementary Figure 3). Jukes–Cantor distance measures species level similarity and a distance < 0.03 was used for potential pathogen classification (Schloss and Handelsman, 2006; Bibby et al., 2010; Chidamba and Korsten, 2015). Among the 38 seed sequences, 9 sequences might come from multiple serotypes of pathogenic *E. coli*, *Shigella flexneri*, *Klebsiella oxytoca*, or *Klebsiella pneumoniae* (Table 1). One sequence (*E. coli*_85263602) was closely related to several strains of *E. coli* O157:H7.

**TABLE 1:** ASV1628 (*E. coli*_unclassified) sequences containing regions within 0.03 Jukes–Cantor distance of known pathogens.

Seed sequences	Closely related pathogens	Regions of distance < 0.03
*E. coli*_105450279	*Escherichia coli* O7:H4 strain MIN14 *Escherichia coli* O78 strain 3	5/10 5/10
*E. coli*_108202198	*Escherichia coli* O80:H26 strain EC-107 *Escherichia coli* O39:NM str. F8704-2 *Escherichia coli* O6:H16 strain M9682-C1	3/12 1/12 3/12
*E. coli*_129173627	*Klebsiella oxytoca* isolate MSB1_10D-sc-2290340	2/12
*E. coli*_154600965	*Shigella flexneri* strain WW1	2/10
*E. coli*_61606331	*Escherichia coli* O157:H16 str. 98-3133	2/12
*E. coli*_85263602	*Escherichia coli* O157:H7 str. 7.1 Anguil *Escherichia coli* O157:H7 str. MB9-1 *Escherichia coli* O157:H7 str. MB41-1 *Escherichia coli* O157:H7 str. 611 *Escherichia coli* O157:H7 str. 2571 *Escherichia coli* O157:H7 str. 7636 *Escherichia coli* O157:H7 str. 2-6-2 *Escherichia coli* O157:H7 str. 3-5-1 *Escherichia coli* O157:H7 str. 1786-2 *Escherichia coli* O157:H7 str. 86-24	3/11 1/12 2/12 4/12 1/12 1/12 1/12 1/12 1/12 1/12
*E. coli*_7800770	*Escherichia coli* O157:H16 str. 98-3133 *Escherichia coli* O89m:H10 str. MIN12	16/24 6/24
*E. coli*_61540546	*Klebsiella pneumoniae* subsp. *Pneumoniae* str. WRC19_AI1572C *Escherichia coli* O7:H4 strain MIN14 *Escherichia coli* O6: H16 strain 2014EL-1346-6	1/13 1/22 2/12

*The ratios (X/Y) in column “Regions of distance < 0.03” indicate that X regions are having a Jukes–Cantor distance of <0.03 with a known pathogen, and Y is the number of total sequenced regions.*

### Bacteria Transmission Routes Identification

**FIGURE 5:**
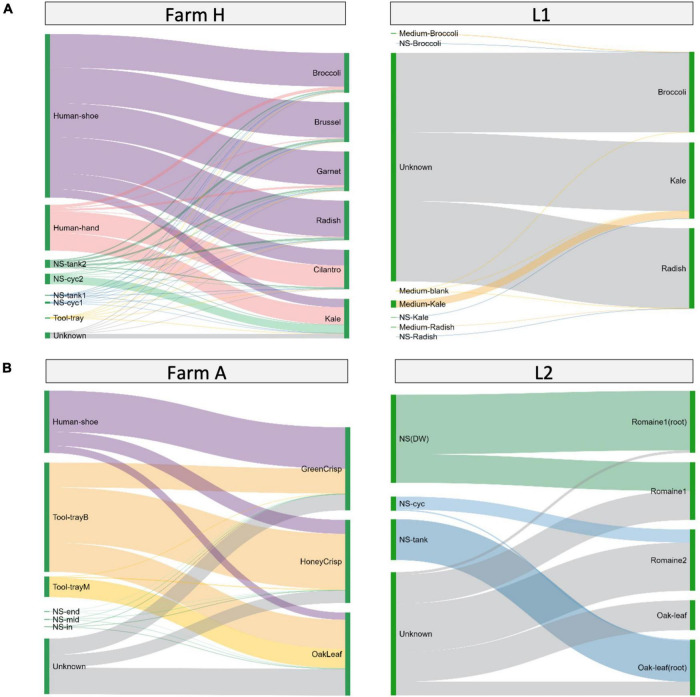
Source environment proportions for hydroponically grown microgreens or lettuces. The samples are grouped by system type of **(A)** microgreens or **(B)** lettuce. Source environment proportions for fresh produce samples estimated using SourceTracker and visualized as Sankey flow diagrams. Environmental source samples are presented on the left and fresh produce samples are “sinks” and presented on the right. The line width of individual flows indicates the percentage contribution of microorganisms from the source to the sink. The contribution proportions from different sources sum up to 100% on the sink sample (right side). The height of individual bars (left side) of source samples represents the sum of proportions to each of the sink samples.

The bacteria transmission route in the hydroponic/aquaponic systems was investigated using SourceTracker and visualized using Sankey flow diagrams (Figure 5). Environmental samples were designated as sources for testing against the bacterial communities from the fresh produce samples. In farm H (Figure 5A left), the worker’s shoes and hands contributed 21.1–89.7% and 1.5–55.1% of the microgreen microbiome, respectively. For farm A (Figure 5B left), worker’s shoe and the baby plant growth tray contributed 8.6–49.8 and 29.9–66.6% lettuce microbiome, respectively. There were 15.2–32.0% of ASVs in lettuce samples, which were not shared with any environmental samples.

When looking at the laboratory systems, L1 microgreens and L2 lettuce had 85.24–99.47% and 47–78% of the ASVs from unknown sources (Figures 5A,B, right). To better understand the source of bacteria, we sampled the microgreen growth medium separately in L1 and the root and edible part in L2. The growth medium or nutrient solution contributed a minor amount of ASVs to microgreens. The nutrient solution reservoir tanks shared large portions (66–96%) of ASVs with the lettuce root and smaller portions (0–46%) with the lettuce leaf. The ASVs from unknown sources could be part of the plant’s background microbiome.

**FIGURE 6:**
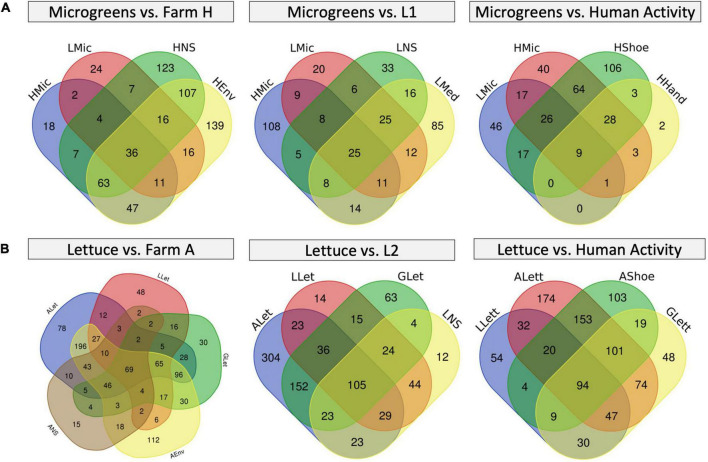
Venn diagrams of microbiome overlapping between different sample types. **(A)** Microgreens overlap with farm H environment (left), with L1 system environment (mid), and farm H worker’s shoes and hands (right). **(B)** Lettuce overlaps with farm A environment (left), L2 system (mid), and farm A worker’s shoes (right). The numbers in each region represent the numbers of unique ASVs in that region.

To take a better look at the plant background microbiome, we constructed Venn diagrams to investigate the microbiome overlaps (Figure 6 and Supplementary Table 7). Farm H microgreens shared 53 ASVs with L1 microgreens and 27 unique ASVs with L1 system environment, while L1 microgreens shared 39 unique ASVs with farm H environment (Figure 6A). In the lettuce systems, there were 141 and 193 overlapping ASVs among all the lettuces (G, farm A, and L2) and between farm A and L2 (Figure 6B). Besides, the L2 lettuce shared 29 unique ASVs with farm A environment, and 46 unique ASVs from farm A lettuce also appeared in the L2 nutrient solution. This result agrees with the observation in beta-diversity that similar fresh produce samples from different systems had shared microbiome patterns. The share ASVs between lettuce samples as well as microgreens mainly belong to genera *Pseudomonas* and *Pantoea*. *Pseudomonas* and *Pantoea* were reported as part of the background microbiome of lettuce leaf (Rastogi et al., 2012). The lettuce samples also shared ASVs from Cyanobacteria that are associated with the water environment.

**FIGURE 7:**
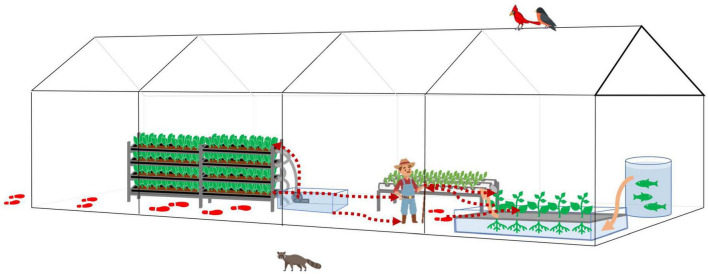
The bacteria transmission affected by human farming activities in a typical greenhouse hydroponic/aquaponic ecosystems. Bacteria transmissions follow the red dotted arrow lines. The footprints indicate the potential contaminants brought in by human activities and transmitted inside the greenhouse. The water flow follows orange solid arrow lines or the water pipes connecting each water body.

Thus, we further proposed a bi-direction bacteria transmission route inside the HCS (Figure 7). With good personnel hygiene, fresh produce would be the major source of bacteria. With poor personnel hygiene, contaminants would enter the system *via* human activities. Tools, water, and workers are vehicles for bacteria transmission, and poor facility hygiene would result in cross-contamination and accumulation of hazards.

## Discussion

The 16s rRNA sequencing, as the gold standard for microbiome profiling, has been used in food production systems to delineate sanitation effectiveness and microbiome shifts (Gu et al., 2019, 2020). However, the 16s rRNA sequencing provides insufficient resolution to distinguish closely related species (UCD Centre for Food Safety et al., 2018; Zwirzitz et al., 2020). 16s-ITS-23s rRNA sequencing together with the ASV taxonomy assignment was reported to provide a higher taxonomy resolution (Martijn et al., 2019; Graf et al., 2021). This study combined site visits, food safety practice survey, traditional plate count, 16S-ITS-23S rRNA sequencing, and multiple bioinformatics tools to investigate the microbiomes, microbial hazards, and bacterial transmission in hydroponic/aquaponic ecosystems. The result gave us a picture of microbial communities and the risk status of the hydroponic/aquaponic ecosystem.

We investigated two types of commercial farm systems (hydroponic and aquaponic) and compared the microbial loads and microbiomes to the laboratory control hydroponic systems. The aerobic plate count measures the overall microbial loads of the circulating water and fresh produce. In literature, the aerobic bacteria count of microgreens and hydroponically grown lettuces was normally in the range of ∼7.5 and ∼4 log CFU/g, respectively, and our samples were within a similar range (Lee et al., 2009; Chandra et al., 2012; Dankwa et al., 2020, 2021). The lettuce from the grocery store had a higher microbial load possibly due to post-harvest procedures (packaging, storage, transportation, and others), which would introduce bacteria and cause bacterial growth (Gil et al., 2015; Dankwa et al., 2020). Farm A lettuce had higher community richness than other lettuce samples possibly due to poor hygiene practice, for example, workers did not switch shoes and clothes between indoor and outdoor farms. Farm A worker’s shoe swab had the highest microbiome richness, and it shared many unique ASVs with farm A lettuce, including ASV833 (*Thermoactinomycetaceae*_unclassified), ASV830 (*Staphylospora*_unclassified), and ASV744 (*Parageobacillus*_unclassified). These ASVs together with ASV1628 (*E. coli*_unclassified) were likely to come from the outside of the greenhouse (Figure 6B and Supplementary Table 7). From these results, we suggested that farm A should invest in personal protection equipment such as changing shoes when working inside the greenhouse.

In hydroponics, the nutrient solution circulates inside the system and links every plant, and it may pose a significant risk of fresh produce cross-contamination (Gil et al., 2015). Farm H nutrient solution exhibited a higher microbial load than other systems, possibly because farm H nutrient solution had a relatively long residing time (1 month). *Pseudomonas* spp. were abundant in farm H. Farm H used protective clothes before entering the greenhouse, which limited the external source of bacteria. *P. aeruginosa* and *P. putida* were reported as the background flora of microgreens that may be present in seeds (Bergšpica et al., 2020). However, *P. fluorescens* (ASV1821) was abundant in farm H and it is one of the major spoilage *Pseudomonas* that cause soft rot and fleshy vegetable tissue (Liao, 2006). Microgreens are harvested by cutting stem above the root and are highly perishable depending on the species (Berba and Uchanski, 2012). Thus, we suggested farm H to include a seed sanitation step in the Standard Operating Procedure, improve the tool sanitation method, and flash the system more often to reduce the microbial hazard accumulation and potentially improve the shelf-life of their microgreen products.

Mapping to the Athena database resulted in many unclassified ASVs. It is probably due to the limitation of database coverage (Graf et al., 2021). For example, in farm A worker’s shoes, 38 sequence reads with >95% similarity were grouped in ASV1628 (*E. coli*_unclassified). In the Athena database, there were 187 strains of *E. coli*, which is just a minimum part of the currently identified *E. coli* strains. To increase classification resolution and confirm pathogenicity, we mapped the 38 seed sequence reads to multiple databases. When using the 16S rRNA databases, some of the sequences were mapped to *Pantoea*, *Enterobacter cloacae* complex, or *Salmonella*. It was because bacteria species may share parts of the 16S rRNA sequence resulted in low taxonomy resolution. By mapping the 16S-ITS-23S sequence read to the bacterial whole genome in NCBI nucleotide collection and using the criteria of Jukes–Cantor distance < 0.03, we could further increase the resolution and identify potential pathogens (Table 1). However, we cannot define a true pathogen using the Jukes–Cantor distance (Bibby et al., 2010). To further confirm the pathogenicity, running different sequencing methods such as WGS on concerned samples would be suggested.

This study showcases that high-throughput 16S-ITS-23S rRNA sequencing can reveal valuable information about the microbiome in hydroponic/aquaponic systems and increase the taxonomy resolution for microbial hazard identification. We pinpointed many ASVs to specific sources and screened for potential pathogens or spoilage organisms in the analyzed systems. Our findings would contribute to understanding the hydroponic/aquaponic system ecology and risk management. Furthermore, the methods used in this study can be applied to other farming/food production systems as a risk assessment tool, as well as deepen our knowledge of microbiomes relationships in ecosystems. Continuing advancements in long-read sequencing strategies of entire rRNA operon and expanding the bacterial gene database coverage will further increase the throughput and taxonomic resolution and may offer a great potential to implement them as a cost-effective tool in microbial hazard identification.

## Data Availability Statement

The datasets presented in this study can be found in online repositories. The names of the repository/repositories and accession number(s) can be found below: https://www.ncbi.nlm.nih.gov/, PRJNA785644.

## Author Contributions

MD designed and performed the experiments, analyzed data, and wrote the manuscript. HF supervised the study and revised the manuscript. Both authors contributed to the article and approved the submitted version.

## Conflict of Interest

The authors declare that the research was conducted in the absence of any commercial or financial relationships that could be construed as a potential conflict of interest.

## Publisher’s Note

All claims expressed in this article are solely those of the authors and do not necessarily represent those of their affiliated organizations, or those of the publisher, the editors and the reviewers. Any product that may be evaluated in this article, or claim that may be made by its manufacturer, is not guaranteed or endorsed by the publisher.

## Footnotes

1
https://rdrr.io/cran/pheatmap/

2
https://sankeymatic.com/build/

3
https://bioinformatics.psb.ugent.be/webtools/Venn/
