# Supplementary material for: Microbial Community Analysis and Food Safety Practice Survey-Based Hazard Identification and Risk Assessment for Controlled Environment Hydroponic/Aquaponic Farming Systems
Source: Front Microbiol. 2022 May 19;13:879260. doi: 10.3389/fmicb.2022.879260 (PMC9161294; doi:10.3389/fmicb.2022.879260)
Supplement: Supplementary file 1 [file Table_1.DOCX]

Supplementary Material


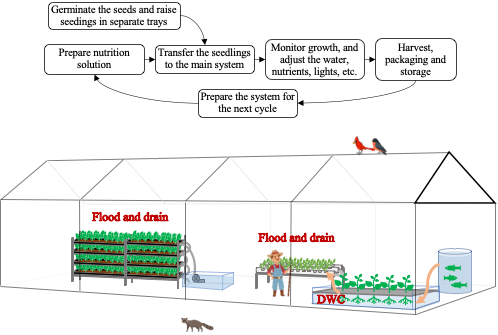


**Supplementary Figure 1.** Hydroponic system set up and operation procedures. The hydroponic system types were labeled in red.

**Supplementary Figure 2.** Rarefaction curves of unrarefied samples.

**Supplementary Figure 3.** 38 sequences in ASV1628 (E. coli_unclassified) and their top 10 most closely related strains.

**Supplementary Table 1.** Sample collected from different systems and used for microbiome analysis

| Source | System | Sample | Amount |
| --- | --- | --- | --- |
| Hydroponic (Farm H) | Flood and drain 1 | Radish microgreens | 25g |
|  |  | Garnet microgreens | 25g |
|  |  | Broccoli microgreens | 25g |
|  |  | Brussels sprout microgreens | 25g |
|  |  | Cilantro microgreens | 25g |
|  | Flood and drain 2 | Kale microgreens | 25g |
|  | Flood and drain 1 | Nutrition solution – reservoir tank | 200mL |
|  |  | Nutrition solution – in-cycle | 100mL |
|  | Flood and drain 2 | Nutrition solution – reservoir tank | 200mL |
|  |  | Nutrition solution – in-cycle | 100mL |
|  | - | Shoe swab 1 | N/A |
|  |  | Shoe swab 2 |  |
|  |  | Hand swab |  |
|  |  | Tray swab |  |
| Aquaponic (Farm A) | DWC | Green oak leaf | 25g |
|  |  | Green crisp | 25g |
|  |  | Honey crisp | 8.3g |
|  |  | Nutrition solution – inlet | 200mL |
|  |  | Nutrition solution – mid | 200mL |
|  |  | Nutrition solution – end | 200mL |
|  | N/A | Shoe swab | N/A |
|  |  | Tray swab (baby plant) |  |
|  |  | Tray swab (mature plant) |  |
| Grocery(G) | (Hydroponic unknown) | Green oak leaf | 25g |
|  | (Hydroponic unknown) | Romaine lettuce | 25g |
|  | (Hydroponic unknown) | Boston Bibb | 25g |
|  | (Hydroponic unknown) | Honey crisp | 25g |
| Lab-grown(L1) | Flood and drain | Kale | 7.5g |
|  |  | Broccoli | 8.1g |
|  |  | Radish | 18.25g |
|  |  | Kale nutrition solution | 200mL |
|  |  | Broccoli nutrition solution | 200mL |
|  |  | Radish nutrition solution | 200mL |
|  |  | Kale growth medium | 0.25g |
|  |  | Broccoli growth medium | 0.25g |
|  |  | Radish growth medium | 0.25g |
|  |  | Blank growth medium | 0.25g |
| Lab-grown(L2) | DWC | Romaine leaf | 10g |
|  |  | Romaine root | 5.5g |
|  |  | Nutrition solution | 150mL |
|  | Flood and drain | Green oak leaf | 25g |
|  |  | Green oak root | 5.4g |
|  |  | Romaine leaf | 20.7g |
|  |  | Nutrition solution-tank | 250mL |
|  |  | Nutrition solution-cycle | 250mL |

**Supplementary Table 2.** Five-section food safety practice survey.

| **Section 1. Equipment, tools, and environment** |
| --- |
| Question 1. How often do you do deep cleaning of the hydroponic system? How?  Question 2. How often do you clean your tools (gloves, scissors, working clothes, buckets, etc.)? How?  Question 3. How often do you clean the floor inside the plant? How?  Question 4. In the past 3 months, any birds or wild animals came inside the facility?  A. Yes, please specify ______ B. No C. I didn’t notice  Question 5. In the past 1 year, any flood water came into the facility?  A. Yes, in the month(s) of ________ B. No |
| **Section 2. Water treatment** |
| Question 1. What water do you use to make the nutrition solution?  A. Tap water B. well water C. surface water D. other water source, please specify______.  Question 2. How do you treat the water before making the nutrition solution?  A. I sanitize it by ________ B. I use the water directly without any pretreatment  Question 3a. Do you circulate the nutrition solution/ irrigation water? (Yes or No)  Question 3b. If answered Yes in the previous question, how often do you replace the nutrition solution?  Question 5. Do you have a sterilization step in the water circulation? A. Yes, how ____ B. No.  Question 6. What nutrients do you use and where do you obtain them? |
| **Section 3. Worker hygiene** |
| Question 1. How many hand washing facilities available on site?  Question 2. Do you wash hands before returning to work? (Yes or No)  Question 3a. Do you have protective clothing and shoes only for working in this facility? (Yes or No)  Question 3b. If answered No in the previous question, how do you treat/ clean your clothes and shoes before entering into the facility?  Question 4. During the last 3 months, have you worked in the facility while you were experiencing diarrhea, cold, flu, vomiting, etc? A. Yes, I experienced ___ for __ times. B. No |
| **Section 4. Food safety awareness** |
| Question 1. Have you received any sanitation training before working in the current farm?  A. Yes, I was trained at _________ B. No  Question 2. Do you have a written food safety plan to follow? (Yes or No)  Question 3. If possible, would you be interested in participating in a farming food safety workshop? (Yes or No) |
| **Section 5. Seed, harvest and preservation** |
| Question 1. Where do you obtain the seeds or seedling?  A. Seed company, _______ B. Harvest from field C. Other, please specify______  Question 2. How do you sanitize the seeds or seedlings before planting?  A. I don’t sanitize them, I plant directly. B. I wash them with __________  C. Other, please specify ________  Question 3. How do you harvest the fruit and vegetables?  A. using bare hand B. hand harvesting, wear gloves  C. smart harvesting machine D. other, please specify_______  Question 4. Please describe after harvesting, how do you sanitize and preserve the produce before selling? |

**Supplementary Table 3.** Five-section food safety practice survey result calculated in scores.

| **Sections (Total available points)** | **Farm_H** | **Farm_A** |
| --- | --- | --- |
| S1. Equipment, tools, environment (10) | 10 | 6 |
| S2. Water treatment (11) | 5 | 5 |
| S3. Worker hygiene (10) | 10 | 9 |
| S4. Food safety awareness (8) | 8 | 4 |
| S5. Seed, harvest, and preservation (9) | 4 | 6 |
| Total score (48) | 37 | 30 |

**Supplementary Table 4.** Prevalence of genera comprising top 50 ASVs

| Genus | Prevalence% | #ASVs in top50 | #ASVs in genus |
| --- | --- | --- | --- |
| Acidovorax | 48 | 3 | 7 |
| Acinetobacter | 47 | 2 | 14 |
| Afipia | 38 | 1 | 3 |
| Bacillaceae_unclassified | 40 | 1 | 1 |
| Bacillus | 73 | 1 | 30 |
| Bacteriovorax | 20 | 1 | 3 |
| Bosea | 53 | 1 | 4 |
| Clostridium | 33 | 1 | 7 |
| Crinalium | 22 | 1 | 1 |
| Curtobacterium | 51 | 1 | 6 |
| Cyanobacteria_unclassified | 76 | 1 | 1 |
| Edaphobacter | 24 | 1 | 1 |
| Enterobacter | 46 | 2 | 5 |
| Exiguobacterium | 40 | 1 | 4 |
| Flavobacterium | 71 | 1 | 8 |
| Granulicella | 20 | 1 | 2 |
| Gryllotalpicola | 33 | 1 | 2 |
| Iamiaceae_Unknown_genus | 42 | 1 | 1 |
| Klebsiella | 22 | 1 | 1 |
| Leptolyngbyaceae_unclassified | 31 | 1 | 1 |
| Massilia | 69 | 1 | 4 |
| Methylobacterium | 47 | 1 | 9 |
| Methylophilus | 24 | 1 | 3 |
| Methylotenera | 44 | 1 | 5 |
| Microbacterium | 41 | 2 | 10 |
| Mycobacterium | 60 | 1 | 4 |
| Oscillatoriophycideae_unclassified | 51 | 1 | 1 |
| Pantoea | 57 | 3 | 10 |
| Paraburkholderia | 13 | 1 | 4 |
| Pseudomonas | 52 | 4 | 48 |
| Rhizobiales_unclassified | 47 | 1 | 1 |
| Rhodocyclales_unclassified | 62 | 1 | 1 |
| Rhodoluna | 18 | 1 | 2 |
| Rubrivivax | 36 | 1 | 2 |
| Scytonema | 29 | 1 | 1 |
| Sphingobacterium | 44 | 1 | 2 |
| Sphingomonas | 47 | 1 | 4 |
| Staphylospora | 13 | 1 | 1 |
| Stenotrophomonas | 49 | 1 | 3 |
| Thermoactinomycetaceae_unclassified | 13 | 1 | 1 |

**Supplementary Table 5.** PERMANOVA for community-level multivariate comparison by sample location and type.

|  | Weighted UniFrac | | Unweighted UniFrac | | Weighted UniFrac | |
| --- | --- | --- | --- | --- | --- | --- |
|  | R^2^ | Pr(>F) | R^2^ | Pr(>F) | R^2^ | Pr(>F) |
| By type | 20.40% | 0.001*** | 18.99% | 0.001*** | 26.09% | 0.001*** |
| By location | 26.47% | 0.001*** | 29.85% | 0.001*** | 25.84% | 0.001*** |
